# Seroprevalence of Varicella-Zoster Virus and Measles among Healthcare Workers in a Tertiary Medical Center in Korea

**DOI:** 10.3390/vaccines10111956

**Published:** 2022-11-18

**Authors:** Ji Hyun Yun, Eunsol Lee, Jeong Hwa Choi, Hyun Kyun Ki, Jiho Park

**Affiliations:** 1 Division of Infectious Diseases, Department of Internal Medicine, Konkuk University Medical Center, Konkuk University School of Medicine, 120-1 Neungdong-ro, Gwangjin-gu, Seoul 05029, Republic of Korea; 2 Department of Infection Control, Konkuk University Medical Center, Seoul 05029, Republic of Korea

**Keywords:** seroprevalence, varicella-zoster, measles, healthcare worker, age, occupation

## Abstract

Measles and varicella still occur in the general population despite the widespread vaccination against them, and healthcare workers (HCWs) are still at risk of exposure to these diseases. Here, we evaluated the seroprevalence of measles and varicella-zoster virus (VZV) in HCWs and the trend of seroprevalence according to age, birth year, and occupational group. The serostatuses of measles and VZV of HCWs during new employee medical examinations between October 2015 and October 2021 were included. Thereafter, the trends of seroprevalence according to age, birth year, and occupational groups were evaluated. Overall, 2070 and 1827 HCWs were evaluated for VZV and measles serostatus, respectively. The seroprevalences of VZV and measles were 91% (1884/2070) and 70% (1284/1827), respectively. Younger HCWs had a significantly lower seroprevalence of measles (*p* = 0.02, age) and VZV (*p* = 0.003, birth year and *p* < 0.001, age). The seroprevalence of measles and VZV was significantly higher among doctors and nursing assistants than among nurses and other HCWs (*p* < 0.001 in both). In conclusion, the seroprevalence of measles and VZV significantly decreased in younger HCWs. Additionally, monitoring the serostatus of measles and VZV and the immunization of susceptible HCWs are required to prepare and control infectious diseases in healthcare facilities.

## 1. Introduction

Varicella and measles were major threats to public health because of their high transmission rates and complications [1,2]. The basic reproduction number of measles and varicella ranges from 13 to 18 and 3.7 to 5.0, respectively [3,4]. The incidence of varicella and measles decreased after the wide application of vaccination [5,6]. In Korea, varicella and measles vaccinations were included in the National Institutes of Health (NIH) program in 1985 and 2005, respectively. However, despite the efficacy of vaccination, varicella and measles still occur worldwide, including in countries with high vaccination rates [7,8,9,10]. Additionally, varicella and measles still occur in Korea, not only in childhood but also in adolescence and younger adults [11,12]. The primary failure of vaccines, secondary failures such as the waning effect of immunity, and the avoidance of vaccination could lead to a new outbreaks of these infectious diseases.

In a previous study that evaluated the seroprevalence of measles in Korea, the seroprevalence declined with time in the general adult population, particularly in those born between 1990 and 2001 [13]. The seroprevalence of measles in healthcare workers (HCWs) also reduced with time and was reported to be between 40% and 90% in HCWs who were born in the 1990s [14,15,16,17,18]. The waning effect of immunity might cause a decrease in the seroprevalence of measles [16]. With a decrease in the seroprevalence of measles, measles outbreaks among HCWs have been reported in some hospitals [19,20].

Seroprevalence of the varicella-zoster virus (VZV) was high and reached over 90% of the general adult population in Korea [21,22]. Recently, one study reported the seroprevalence of measles, mumps, rubella, and VZV in nurses in a tertiary hospital [15]. Although the seroprevalence of VZV is approximately 90% in all nurses, the seroprevalence of VZV significantly declined to 86% in young nurses. Previous studies that evaluated the seroprevalence of VZV in HCWs were limited by the small number of older age people and a limited population.

In this study, we evaluated the serostatuses of measles and VZV among HCWs, including doctors, nurses, nurse assistants, and other healthcare givers. The seroprevalence of measles and VZV and its trends were assessed according to birth year, age, and occupational groups.

## 2. Materials and Methods

### 2.1. Study Population

This study included HCWs who were examined for the serostatuses of measles and VZV during new employee medical checkups at Konkuk University Medical Center, a tertiary hospital in Korea, between October 2015 and October 2021. The seroprevalence of measles and VZV in HCWs was assessed according to their birth year, age, and occupational groups. Birth years were distributed in 5-year intervals; 1950–1954, 1955–1959, 1960–1964, 1965–1969, 1970–1974, 1974–1979, 1980–1984, 1984–1989, 1990–1994, and 1995–1999. Age groups were also distributed in 5-year intervals; 20–24, 25–29, 30–34, 35–39, 40–44, 45–49, 50–54, 55–59, and over 60. Age was calculated by the difference between the year of the test performed and the birth year. Additionally, the occupations of HCWs were grouped into doctors, nurses, nurse assistants, and other HCWs. The seroprevalence of VZV and measles was calculated as the seropositivity rate in each group. Thereafter, the changes in seroprevalence according to birth year and age groups and the comparison of seroprevalence between occupation groups were evaluated. The study was approved by the Institutional Review Board of the Konkuk University Medical Center (IRB No. 2022-08-069).

### 2.2. Changes in New Employee Medical Checkups

Recently hired HCWs were examined for their immunity to measles, VZV, hepatitis B, and hepatitis C, and the presence of latent tuberculosis as a new employee medical checkup. Only immunity to hepatitis B was assessed when the first hospital originally opened. The evaluations of immunity to VZV and measles were included in the new employee medical checkups in March 2009 and March 2016, respectively. The serostatus of measles was evaluated in HCWs who did not prove to have immunity to measles by providing a certificate of two-dose vaccination or previous infection. VZV serostatus was assessed to determine VZV immunity. Vaccinations for susceptible infectious diseases were recommended to HCWs who were susceptible to measles, VZV, and hepatitis B.

### 2.3. Evaluation of the Serostatus

The serostatus of measles was evaluated using a chemiluminescent immunoassay (LIAISON, DiaSorin, Italy). The test result was regarded as positive when the titer was equal to or above 16.5 AU/mL, negative when the titer was below 13.5 AU/mL, and indeterminate when the titer was between 13.5 and 16.5 AU/mL. The serostatus of VZV was evaluated by an enzyme-linked immunosorbent assay using a Chorus analyzer (DIESSE Diagnostica Senese, Siena, Italy). Additionally, the result was considered positive when the titer was above 1.2, negative when the titer was below 0.8, and indeterminate when the titer was between 0.8 and 1.2. The serostatuses of measles and VZV were examined according to the manufacturer’s instructions [23,24]. Positive antibody results were considered seropositive.

### 2.4. Statistical Analysis

The seroprevalence of VZV and measles was calculated among the overall population, birth year groups, age, and occupational groups. A correlation analysis was used to evaluate the correlation between the seroprevalence of VZV and measles according to birth year and age. Furthermore, a linear regression analysis was used to assess the trend of seroprevalence according to birth year and age. Comparison of the seroprevalence of measles and VZV between occupational groups was evaluated by Chi-square and Fisher’s exact tests. Moreover, comparisons of the seroprevalence between each occupational group were conducted. Statistical significance was considered when the *p*-value was < 0.05. In the comparison of seroprevalence between each occupation group (e.g., doctors vs. nurses), statistical significance was considered when the *p*-value was < 0.008 (according to Bonferroni’s method). Statistical analyses were conducted using SPSS (version 22.0; SPSS, Inc., Chicago, IL, USA), and graphs were obtained using PRISM-5 (GraphPad Software, San Diego, CA, USA).

## 3. Results

### 3.1. Study Population

In this study, overall, 2070 HCWs were examined for the serostatus of VZV, of which 1 (0.1%) person was born in 1950–1954, 4 (0.2%) in 1955–1959, 15 (0.7%) in 1960–1964, 29 (1.4%) in 1965–1969, 20 (1.0%) in 1970–1974, 28 (1.4%) in 1975–1979, 140 (6.8%) in 1980–1984, 350 (16.9%) in 1985–1989, 908 (43.9%) in 1990–1994, and 575 (27.8%) in 1995–1999. According to the age group, the population distribution was as follows: 699 (33.8%) in 20–24 years, 867 (41.9%) in 25–29 years, 309 (14.9%) in 30–34 years, 113 (5.5%) in 35–39 years, 17 (0.8%) in 40–44 years, 17 (0.8%) in 45–49 years, 29 (1.4%) in 50–54 years, 15 (0.7%) in 55–59 years, and 4 (0.2%) in over 60 years.

Overall, 1827 HCWs were assessed for the serostatus of measles, of which 1 (0.1%) was born in 1950–1954, 4 (0.2%) in 1955–1959, 15 (0.8%) in 1960–1964, 29 (1.6%) in 1965–1969, 19 (1.0%) in 1970–1974, 27 (1.5%) in 1975–1979, 134 (7.3%) in 1980–1984, 330 (18.1%) in 1985–1989, 836 (45.8%) in 1990–1994, and 432 (23.7%) in 1995–1999. According to the age group, population distribution was as follows: 573 (31.4%) in 20–24 years, 780 (42.7%) in 25–29 years, 288 (15.8%) in 30–34 years, 106 (5.8%) in 35–39 years, 16 (0.9%) in 40–44 years, 16 (0.9%) in 45–49 years, 29 (1.6%) in 50–54 years, 15 (0.8%) in 55–59 years, and 4 (0.2%) in over 60 years.

### 3.2. Seroprevalence of VZV

The seroprevalence of VZV was 91% (90–92%, 95% confidence interval [CI]), and the seroprevalence was 100% in HCWs who were born before 1965 (Table 1). In most birth year groups, seroprevalence was over 90%, except for 1995–1999 (89%). Additionally, seroprevalence was 100% in HCWs who were above 55 years old and lowest in HCWs who were less than 30 (90%) years old. HCWs with seronegative VZV were 87 (4%) and were only observed in HCWs who were born after the 1980s and were younger than those in their 40s.

### 3.3. Seroprevalence of Measles

The seroprevalence of measles was 70% (68–72%, 95% CI). The seroprevalence of the birth year groups was highest in 1950–1954 (100%) and lowest in 1975–1979 (63%) (Table 2). In most birth year groups, seroprevalence was lower than 90%, except for in 1950–1954 and 1965–1969 birth year groups. Moreover, seroprevalence was 100% in patients aged > 60 years and lowest in patients aged 20–24 (56%). In most age groups, seroprevalence was lower than 90%, except for the over 60 and 50–54 age groups. Seronegative HCWs were 296 (16%) and were only observed in HCWs who were born after the 1970s and were younger than those in their 50s.

### 3.4. Seroprevalence of Measles and VZV According to Occupational Group

The seroprevalence of VZV and measles was highest in nursing assistants (100% for VZV and 92% for measles) and lowest in nurses (91% for VZV and 68% for measles) and other HCWs (87% for VZV and 68% for measles) (Table 3). The occupational groups showed significant differences in the seroprevalence of measles and VZV (*p* < 0.001 in both). The seroprevalence of measles and VZV was significantly higher in doctors than in nurses (*p* = 0.002 for measles and *p* = 0.004 for VZV) or other HCWs (*p* = 0.005 for measles and *p* < 0.001 for VZV). Additionally, nurse assistants showed a higher seroprevalence of measles than nurses (*p* = 0.002) and other HCWs (*p* = 0.002). There were no significant differences between the seroprevalence of doctors and nurse assistants.

### 3.5. Trends of Seroprevalence of Varicella-Zoster and Measles

**Figure 1:**
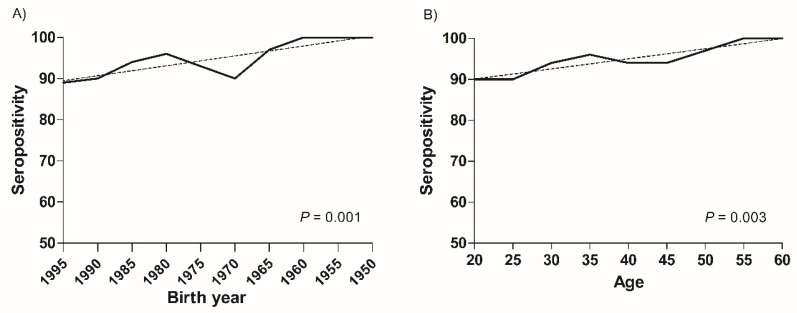
Changes of seroprevalence of varicella-zoster virus in healthcare workers according to birth year and age groups. The seroprevalence of varicella-zoster virus in healthcare workers significantly decreased according to (**A**) the birth year groups (*p* = 0.001) and (**B**) the age groups (*p* = 0.003).

**Figure 2:**
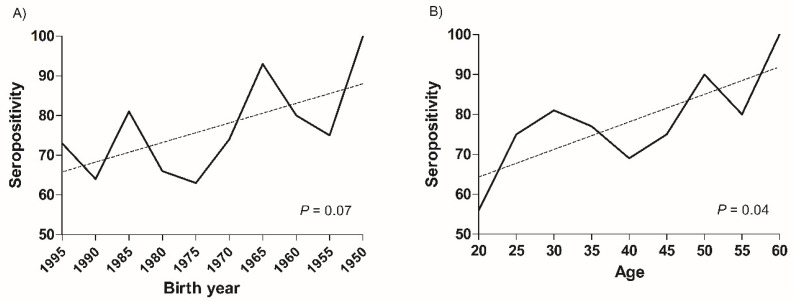
Changes of seroprevalence of measles in healthcare workers according to birth year and age groups. The seroprevalence of measles in healthcare workers decreased according to (**A**) the birth year groups (*p* = 0.07) and (**B**) significantly decreased according to the age groups (*p* = 0.04).

The seroprevalence of VZV significantly correlated with the birth year (*p* = 0.001) and age (*p* = 0.003). In the linear regression analysis, the seroprevalence of VZV significantly decreased in younger HCWs (*p* = 0.003, birth year and *p* < 0.001, age) (Figure 1). Furthermore, the seroprevalence of measles significantly correlated with age (*p* = 0.04) rather than birth year (*p* = 0.07), and the seroprevalence of measles was reduced in younger HCWs. This trend was significant in the age group (*p* = 0.02) rather than in the birth year group (*p* = 0.06) (Figure 2).

## 4. Discussion

In this study, the seroprevalence of VZV was 91%. Although the seroprevalence of VZV was above 90% in most age groups, the seroprevalence in the younger age group was lower than 90%. The seroprevalence of VZV significantly decreased with age. Moreover, the seroprevalence of measles was 70%, which was lower than 90% in most age groups. The seroprevalence of measles also significantly decreased with younger age. Doctors and nurse assistants had significantly higher seroprevalence than nurses and other HCWs.

The incidence of measles has decreased with measles vaccination [6]. However, nosocomial measles infections occur in many countries and threaten HCWs and patients, especially those who are immunocompromised [25]. In Korea, measles elimination was declared in 2006 with a two-dose measles vaccination, and the vaccination rate was >95% [26]. However, measles outbreaks have been reported in schools, healthcare facilities, and travelers to endemic countries [12]. Similar to measles, the incidence of varicella significantly decreases with vaccination [5,8]. In Korea, one dose of varicella vaccination was included in the NIH in 2005 [27]. Subsequently, the vaccination rate among elementary school entrance-aged children reached 93% in a study conducted in 2013 [28]. However, despite the high childhood vaccination rate, varicella incidence has been increasing [29]. Recently, the incidence of measles and varicella has decreased with the outbreak of coronavirus disease 2019 because of the social distancing [11,30]. In Korea, measles cases were 194 in 2019, six in 2020, and zero in 2021 and varicella cases were 82,868 in 2019, 31,430 in 2020, and 20,929 in 2021. However, as outbreak of coronavirus disease 2019 has decreased, social distancing has been reduced or halted in many countries. Because of concerns about the outbreak of transmissible infectious diseases, such as measles and VZV, after social distancing is halted, monitoring of immunity in HCWs and proper vaccination should be considered [31,32].

The present study showed 91% seropositivity for VZV among the HCWs. Our finding was similar to previous studies that reported a seroprevalence of over 90% in Korean adults [15,21,22,33]. The seroprevalence of VZV was higher than 90% in other countries, including Finland, Denmark, Japan, and Singapore [34,35,36,37]. These results could meet the population immunity threshold for varicella [38]. However, seroprevalence significantly decreased in younger HCWs in the present study, and this trend was also observed in another study conducted in Korea [15]. Furthermore, the seroprevalence of VZV in the younger population was lowered in Singapore [34]. When this trend was maintained, the seroprevalence of VZV decreased below the population immunity threshold. Conversely, this might have caused an improvement in the seroprevalence of VZV because the varicella vaccination in the NIH program was included in 2005. However, uncertainties still exist because breakthrough infections have been reported, especially in one dose-vaccinated population [9,39,40,41]. Therefore, continuous evaluation of the seroprevalence of VZV is required to determine the policies of healthcare facilities. Furthermore, a change in vaccination strategy from one dose to a two-dose vaccination should be considered to improve vaccine effectiveness.

The present study showed that the seroprevalence of measles was 70% and the seropositive rate was variable from 55% to 100% with age group. This variation in seropositive rate could result from the changes in vaccine strategy and the incidence of measles in Korea. Previous studies similarly reported the seroprevalence of measles from 73% to 93% among HCWs [14,15,16,17,18]. The seroprevalence of measles was lower than the threshold of population immunity and significantly lower in younger adults than in older adults, and this is one of the causes of the recent measles outbreaks in HCWs. In a meta-analysis conducted in Europe, the seroprevalence of measles decreased in HCWs born after 1980 [42]. Furthermore, studies conducted in Finland, Denmark, and Japan similarly reported a decline in the seroprevalence of measles in younger HCWs [35,36,37]. In Korea, measles vaccination was included in the NIH program in 1985, and a two-dose vaccination was initiated in 1995. In 2001, a catch-up vaccination was conducted for children born between 1986 and 1995 owing to the measles outbreak. Despite the two-dose vaccination of measles and the high vaccination rate, the seroprevalence of measles lowered at a younger age in HCWs. To enhance the immunity to measles, a third vaccination against measles might be needed. Some studies reported enhanced immunity for measles after the third vaccination [43,44,45]. Furthermore, seroconversion occurred in 99% of seronegative HCWs who received catch-up vaccination for measles [14]. However, more studies are needed to assess the adequate target, effectiveness, adverse effects of the third vaccination, and cost-effectiveness. The evaluation of measles immunity might still be helpful among HCWs because of the low seroprevalence of measles.

The seroprevalence of measles and VZV was significantly different between occupational groups. In previous studies, the seroprevalence of measles and VZV were significantly different between the occupational groups [14,46]. However, statistical significance was not observed when the multivariable analysis was conducted, including age, sex, occupation, and vaccination history. Because we could not evaluate the vaccination history of HCWs, an important factor of seroprevalence, the explanation could be limited. However, the mean ages of the doctors, nurses, nurse assistants, and other HCWs were 32, 25, 51, and 28, respectively, which were significantly different. Given that the present study showed the decline of seroprevalence of measles and VZV with younger age, relatively younger age of nurses and other HCWs than doctors and nurse assistants could have led to lower seroprevalence of those occupational groups.

The present study had some limitations. First, the effect of the NIH program on the seroprevalence of VZV among HCWs could not be evaluated. Therefore, continuous evaluation of seroprevalence among HCWs is required to assess the varicella vaccination effectiveness. Second, vaccination history was not evaluated. This study was conducted with new employee medical examinations; hence, we could not evaluate the vaccination history of HCWs. Considering that the vaccination rate of measles is >90% in the general population, measles vaccination history might have little effect on seroprevalence. Additionally, because we could not evaluate the varicella vaccination history, overestimation or underestimation of the seroprevalence of VZV could occur. However, the overall seroprevalence of VZV in this study was similar to that reported in previous studies. Third, because the present study was performed among HCWs, a selection bias could have influenced the results. Seroprevalence could be overestimated in HCWs compared with the general population. Fourth, the study was conducted at one medical center. Therefore, extrapolation to other geographical areas and healthcare facilities is limited. Fifth, the older age groups over the 50s were relatively small. Seroprevalence was evaluated during the new employee medical examination. Therefore, the older age groups were relatively smaller than the younger ones. However, the seroprevalence of measles and VZV significantly decreased in younger HCWs.

## 5. Conclusions

The seroprevalence of measles and VZV among the HCWs was 70% and 91%, respectively. The seroprevalence of measles and VZV was significantly lower in the younger HCWs than the older ones. Additionally, nurses and other HCWs had a lower seroprevalence of measles and VZV than doctors and nursing assistants. Continuous monitoring of seroprevalence is required to modify the infection control strategy and prevent outbreaks of infectious diseases in healthcare facilities.

## Figures and Tables

**Table 1:** Immunologic status of the varicella-zoster virus of healthcare workers according to birth year and age group.

	Total	Positive	Indeterminate	Negative
Birth year				
1950–1954	1	1 (100)	0	0
1955–1959	4	4 (100)	0	0
1960–1964	15	15 (100)	0	0
1965–1969	29	28 (97)	1 (3)	0
1970–1974	20	18 (90)	2 (10)	0
1975–1979	28	26 (93)	2 (7)	0
1980–1984	140	135 (96)	1 (1)	4 (3)
1985–1989	350	329 (94)	10 (3)	11 (3)
1990–1994	908	816 (90)	49 (5)	43 (5)
1995–1999	575	512 (89)	34 (6)	29 (5)
Age				
>60	4	4 (100)	0	0
55–59	15	15 (100)	0	0
50–54	29	28 (97)	1 (3)	0
45–49	17	16 (94)	1 (6)	0
40–44	17	16 (94)	1 (6)	0
35–39	113	108 (96)	3 (3)	2 (2)
30–34	309	291 (94)	8 (3)	10 (3)
25–29	867	780 (90)	47 (5)	40 (5)
20–24	699	626 (90)	38 (5)	35 (5)
Total	2070	1884 (91)	99 (5)	87 (4)

Data are no. (%) of healthcare workers, unless otherwise indicated.

**Table 2:** Immunologic status of measles of healthcare workers according to birth year and age group.

	Total	Positive	Indeterminate	Negative
Birth year				
1950–1954	1	1 (100)	0	0
1955–1959	4	3 (75)	1 (25)	0
1960–1964	15	12 (80)	3 (20)	0
1965–1969	29	27 (93)	2 (7)	0
1970–1974	19	14 (74)	3 (16)	2 (11)
1975–1979	27	17 (63)	7 (26)	3 (11)
1980–1984	134	89 (66)	25 (19)	20 (5)
1985–1989	330	268 (81)	46 (14)	16 (5)
1990–1994	836	537 (64)	135 (16)	164 (20)
1995–1999	432	316 (73)	25 (6)	91 (21)
Age				
>60	4	4 (100)	0	0
55–59	15	12 (80)	3 (20)	0
50–54	29	26 (90)	3 (10)	0
45–49	16	12 (75)	2 (13)	2 (13)
40–44	16	11 (69)	3 (19)	2 (13)
35–39	106	82 (77)	16 (15)	8 (8)
30–34	288	234 (81)	35 (12)	19 (7)
25–29	780	583 (75)	91 (12)	106 (14)
20–24	573	320 (56)	94 (16)	159 (28)
Total	1827	1284 (70)	247 (14)	296 (16)

Data are no. (%) of healthcare workers, unless otherwise indicated.

**Table 3:** Seroprevalence of measles and varicella-zoster virus according to the occupational group.

	Seroprevalence of Varicella-Zoster Virus	*p* Value	Seroprevalence of Measles	*p* Value
Occupational group		<0.001		<0.001
Doctors	449/473 (95) ^a,b^		318/417 (76) ^a,b^	
Nurses	1013/1118 (91) ^a^		641/943 (68) ^a,c^	
Nursing assistants	39/39 (100)		35/38 (92) ^c,d^	
Other HCWs	383/440 (87) ^b^		290/429 (68) ^b,d^	

Data are presented seropositive no./total no. (%) of each occupational group. Abbreviations: HCW; healthcare workers. ^a^ Significantly different (*p* < 0.008) between doctors and nurses. ^b^ Significantly different (*p* < 0.008) between doctors and other HCWs. ^c^ Significantly different (*p* < 0.008) between nurses and nursing assistants. ^d^ Significantly different (*p* < 0.008) between nursing assistants and other HCWs.

## Data Availability

Data sharing is not applicable to this article.
